# Fusion of PspA to detoxified pneumolysin enhances pneumococcal vaccine coverage

**DOI:** 10.1371/journal.pone.0291203

**Published:** 2023-12-14

**Authors:** Barbara Milani, Tanila Wood dos Santos, Maria Eduarda Souza Guerra, Sheila Oliveira, Cibelly Goulart, Greiciely O. André, Luciana C. C. Leite, Thiago R Converso, Michelle Darrieux

**Affiliations:** 1 Laboratório de Microbiologia Molecular e Clínica, Universidade São Francisco, Bragança Paulista, Brazil; 2 Programa de Pós-Graduação Interunidades em Biotecnologia-USP-IPT-IB, São Paulo, Brazil; 3 Centro de Biotecnologia, Instituto Butantan, São Paulo, Brazil; BOKU: Universitat fur Bodenkultur Wien, AUSTRIA

## Abstract

Despite the implementation of conjugate vaccines in several countries, *S*. *pneumoniae* continues to pose a great burden worldwide, causing around 1 million annual deaths. Pneumococcal proteins have long been investigated as serotype-independent vaccines against this pathogen, with promising results. However, it is a consensus that one antigen alone will not be sufficient to provide long-term protection with wide coverage. Amongst the most well studied pneumococcal proteins are PspA and pneumolysin (Ply), two major virulence factors required by the bacterium for successful invasion of host tissues. PspA is highly immunogenic and protective, but it is structurally variable; pneumolysin is conserved among different pneumococci, but it is toxic to the host. To overcome these limitations, N-terminal PspA fragments have been genetically fused to non-toxic pneumolysin derivatives (PlD) to create PspA_PlD chimeras. Mouse immunization with these fusions confers protection against pneumococcal strains expressing heterologous PspAs, which correlates with antibody-induced complement C3 deposition on the surface of multiple pneumococcal strains. Analysis of mutant strains lacking PspA or Pneumolysin shows that both proteins contribute to the antibody-mediated enhancement in complement deposition induced by the fusion. These results expand previous data evaluating PspA_PlD and demonstrate that the fusion combines the protective traits of both proteins, inducing antibodies that efficiently promote complement deposition on multiple strains and cross-protection.

## Introduction

*Streptococcus pneumoniae* is an opportunistic pathogen that colonizes the nasopharynx and oropharynx of healthy individuals. Although colonization is commonly asymptomatic, under certain conditions it may progress to local or systemic diseases; which classifies this microbe as the second most common cause of bacterial mortality, responsible for one of the greatest problems of public health worldwide [1, 2].

The current vaccines used in prophylaxis against pneumococcal diseases are based on capsular polysaccharides conjugated with carrier proteins which, although effective against invasive infections, tend to lose efficacy overtime due to serotype replacement [3, 4]. The conjugate vaccines have high production costs, which further limit their implementation in developing countries, where the disease burden is highest [3]. Thus, protein-based, serotype independent vaccines emerge as a promising alternative to provide greater coverage at reduced costs [5].

Pneumococcal surface protein A (PspA) and Pneumolysin are among the top candidates to be included in protein vaccines against *S*. *pneumoniae* (revised in [6]). In particular, the combination of these proteins is protective against infection with different pneumococcal isolates [7–11]. Previous work from our group evaluated the immunogenicity and protective efficacy of hybrid vaccines containing the N-terminal region of PspA fused to detoxified pneumolysin (PlD) mutants [12]. In that study, the chimeric protein rPspA1_PlD1 was able to protect mice against lethal challenge with two pneumococci of different serotypes expressing PspAs of family 1. Protection was associated with antibody-mediated C3 deposition on the bacterial surface, and increased opsonophagocytosis of antibody-coated pneumococci by mouse peritoneal cells.

Despite its high immunogenicity and prevalence among clinical isolates of pneumococci, PspA exhibits structural and serological variability, especially in the N-terminal, exposed half of the protein [13], which could limit the efficacy of PspA-based vaccines. Analysis of the sequence variations in PspA identified a domain including 100 aminoacids within the N-terminal half of the molecule, named clade-defining region, which was used to classify PspAs in three families and 6 clades. Families 1 and 2 (clades 1 to 5) are present in most clinical isolates [13, 14]. Different PspAs exhibit variable degrees of cross-reactivity, which roughly follow the levels of similarity among the aminoacid sequences; however, studies investigating the cross reactivity of different molecules within each major PspA family found great variations, with a few sequences being more cross-reactive than others [15, 16]. Based on those studies, we have selected a clade 1 PspA that induced the production of antibodies with the greatest cross-reaction among heterologous molecules, for inclusion in the chimeric protein formulation. To test the level of cross-reactivity and cross-protection induced by rPspA1_PlD1, we evaluated the protective efficacy of the vaccine against infection with pneumococcal strains bearing heterologous PspAs; the mechanisms underlying cross-protection were determined, as well as the contribution of each individual protein to the protection conferred by the chimera.

## Materials and methods

### Bacterial strains and growth conditions

The pneumococcal strains used in this work are shown in Table 1. The bacteria were kept as frozen stocks (-80 ºC) in Todd-hewitt medium supplemented with 0,5% yeast extract (THY) and 15% glycerol; when necessary, the bacteria were thawed, plated on blood agar and incubated at 37 ºC overnight in microaerophilic conditions. On the next day, the colonies were transferred to liquid THY medium and cultured until they reached an optical density at 600 nm between 0.4–0.5, corresponding to approximately 10^6^ CFU/ml.

**Table 1:** Pneumococcal strains used in this study.

Strain	Serotype	PspA Clade	Source	Reference
245/00	14	1	IAL	[16]
St P1153	9V	3	UFG	[14]
3JYP2670	3	4	UAB	[17]
St P490	14	4	UFG	[14]
St 255/00	6A	5	UFG	[14]
St P865	23F	5	UFG	[14]
D39^#^	2	2	UAB	[18]
ΔPspA *^#^	2	-	UAB	[19]
D39_ΔPly^#^	2	2	UAB	[20]

IAL: Instituto Adolfo Lutz, São Paulo, Brazil

UAB: University of Alabama at Birmingham

UFG: Universidade Federal de Goiás, Goiânia, Brazil

*ΔPspA is a mutant PspA negative strain derived from D39.

^#^ D39, ΔPspA and D39_ ΔPly were kindly provided by Dr Anders Hakansson from Lund University in Malmö, Sweden.

### Recombinant proteins

A gene fragment encoding the N-terminal region of *pspA* clade 1 and the proline-rich domain, was amplified from the genomic DNA of strain St 245/00, cloned into pAE-6xHis vector and expressed in *E*. *coli* as described previously [16]. PlD1 was obtained through site directed mutagenesis of the *ply* gene from D39 strain by PCR–according to the method proposed by Nelson and Long (1989) [21] and Ho *et al* (1989) [22] and contains a His-Arg substitution at position 367, as described in [12]. PspA1-PlD1 hybrid was produced by genetic fusion of *pspA1* and *plD1* through their cohesive ends. The *plD*1 gene fragment amplified by PCR was first ligated into pGEMT-easy vector (Promega), generating pGEMT-easy_plD1. Next, *plD1* was cut out of the vector by endonuclease digestion (using the restrictions enzymes *Xho*I and *Eco*RI–Thermo Fisher, whose target DNA sequences had been inserted in the primers for *plD*1 amplification) and ligated into pAE-*pspA* which had been previously digested with the same endonucleases. The final product was pAE_*pspA-plD1*. A schematic representation of the final construct can be found in S1 Fig. The recombinant DNA was expressed in competent *E*. *coli BL21DE3* cells and purified through Nickel affinity chromatography.

### Animals and immunization

All experiments employing mice have been approved by the ethics committee at Universidade São Francisco, Bragança Paulista–SP (CEUAUSF, permit n 001.08.12). Female BALB/c mice from CEMIB (Campinas, Brazil) were immunized subcutaneously with 3 doses of 8 μg of rPspA1, 11.2 μg of rPlD1, 20 μg of co-administered proteins or 20 μg of the hybrid PspA1-PlD1 at 10-day intervals, diluted in 0.9% saline solution with 50 μg of Al(OH)3 as adjuvant. The adjuvant alone was used as a control. Two weeks after the last immunization, blood was collected from the retro-orbital plexus, centrifuged at 500 x g for 10 minutes, and serum was stored at -20ºC.

### Antibody binding and antibody-mediated complement deposition

Antibody binding and complement deposition were performed according to the protocols described by Goulart et al (2013) [12] and Converso et al 2017 [23]. Briefly, pneumococcal strains expressing family 2 PspAs (Table 1) were grown in THY up to the mid-log phase, washed with PBS, and incubated in the presence of heat-inactivated pooled sera from mice immunized with the recombinant proteins at a final concentration of 5%. Following another wash, the samples were incubated with 100 μL of PBS containing FITC-conjugated anti-mouse IgG (MP Biomedicals) at 1:1000 dilution, washed two more times with PBS, resuspended in 1% formaldehyde and analyzed by flow cytometry, using FACS Canto II (BD Biosciences).

For the complement deposition assay, after incubation with antisera, the samples received 10% of BALB/c NMS (normal mouse serum) as a complement source and were incubated at 37 ºC for another 30 min. The samples were washed two times with PBS and incubated with FITC-conjugated anti-C3 (MP Biomedicals) at a 1:500 dilution in 100 μL of PBS on ice. Complement deposition was also evaluated in presence of anti-PspA1_PlD1 antibodies in *S*. *pneumoniae* D39 and its isogenic mutants lacking PspA (ΔPspA) or Ply (D39_ΔPly), using the same protocol.

### Challenge

Two weeks after the last immunization, the animals were challenged intravenously with 10^6^ CFUs of *S*. *pneumoniae* strain 3JYP2670 diluted in PBS to a final volume of 50 μl per mouse. The animals were monitored for 12 days, and moribund mice were euthanized by anesthesia using 300 mg/kg of ketamine and 30 mg/kg of xylazine (ten times the dose necessary to anesthetize the mice). At the endpoint, all surviving animals were euthanized.

### Statistical analysis

For the antibody binding experiments, statistical analysis was performed using one way ANOVA with Dunnet’s posttest for comparison of multiple groups against the control, or Student t test for comparison between two groups, when indicated. For the complement deposition assays, statistical analysis was performed using ANOVA with Tuckey’s posttest for comparison of multiple groups, or Student t test for comparison between two groups, when indicated. In both cases, values of p ≤ 0,05 were considered statistically significant.

For the challenge experiments, the survival times in immunized and control groups were compared using log rank test. p values ≤ 0,05 were considered statistically significant.

## Results

### Antibody binding onto pneumococcal surface

**Fig 1:**
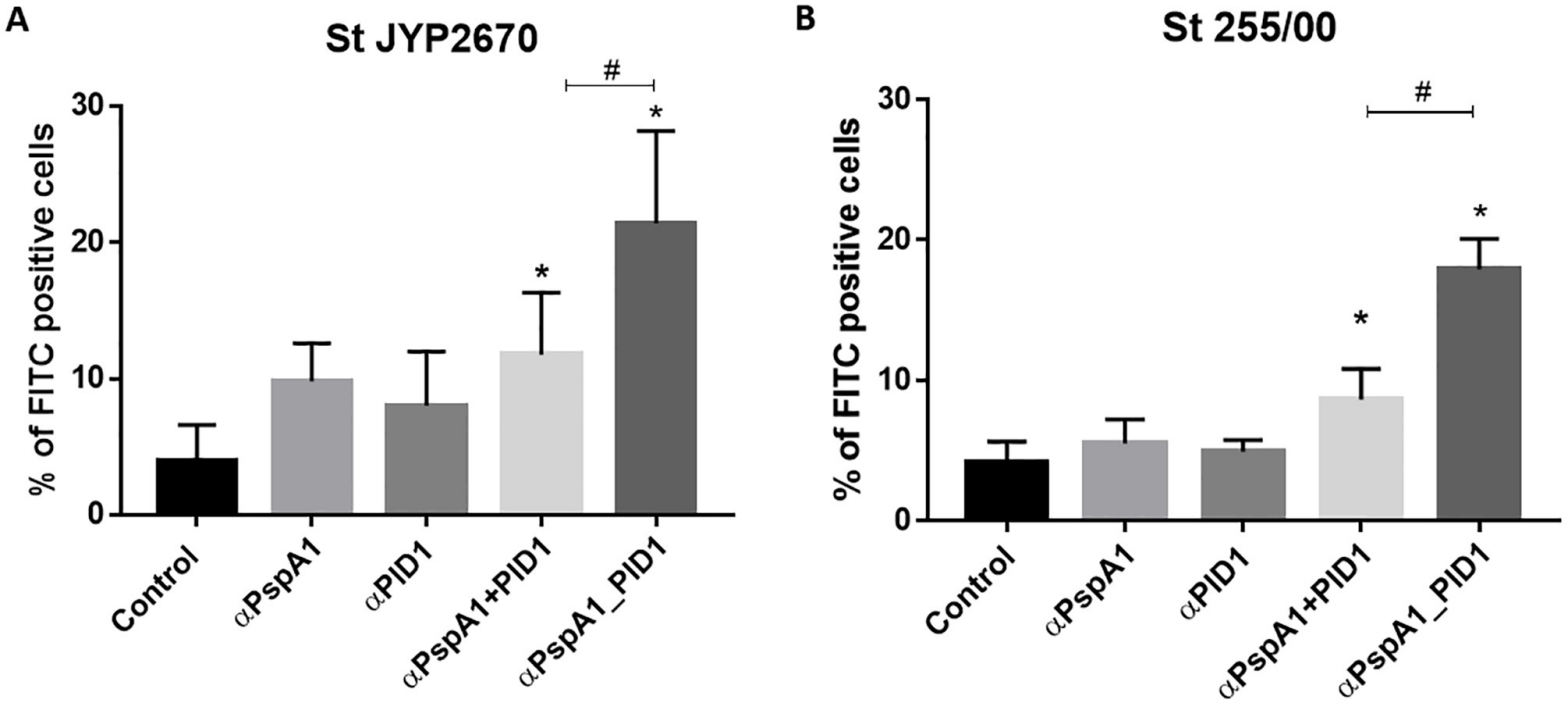
Antibody binding on the surface of *Streptococcus pneumoniae*. Pneumococcal strains 3JYP2670 (A) and St 255/00 (B) were treated with serum from mice immunized with PspA1, PlD1, the proteins mixed, the fusion PspA1_PlD1 or adjuvant alone (control) and FITC conjugated anti-mouse IgG. The percentage of FITC positive bacteria (representing antibody binding) is shown for each group. The error bars represent the standard deviation of the replicates from the mean percentage of FITC positive cells. Statistical analysis was performed using ANOVA with Dunnet’s posttest. *p≤0,05 in comparison with the control; ^#^ p≤0,05 when comparing the proteins mixed and fused.

The ability of antibodies against the recombinant proteins to recognize and bind to the native proteins on the bacterial surface was analyzed by flow cytometry using pneumococcal strains 3JYP2670 and St 255/00, which express PspAs from family 2, clades 4 and 5, respectively. Antisera induced by immunization with the hybrid, PspA_-PlD1, exhibited strong binding to both pneumococcal strains, with a percentage of FITC positive bacteria significantly superior to the control (Fig 1). The group injected with the co-administered proteins also presented an increased binding to the pneumococci, but the percentage of positive cells was lower than those incubated with antibodies against the hybrid. In the case of St 255/00, the proportion of positive bacteria after incubation with anti-PspA1_PlD1 was twice as high as that of the co-administered proteins. Sera from mice injected with the isolated proteins, on the other hand, did not bind efficiently to the pneumococci.

### Antibody mediated complement deposition

**Fig 2:**
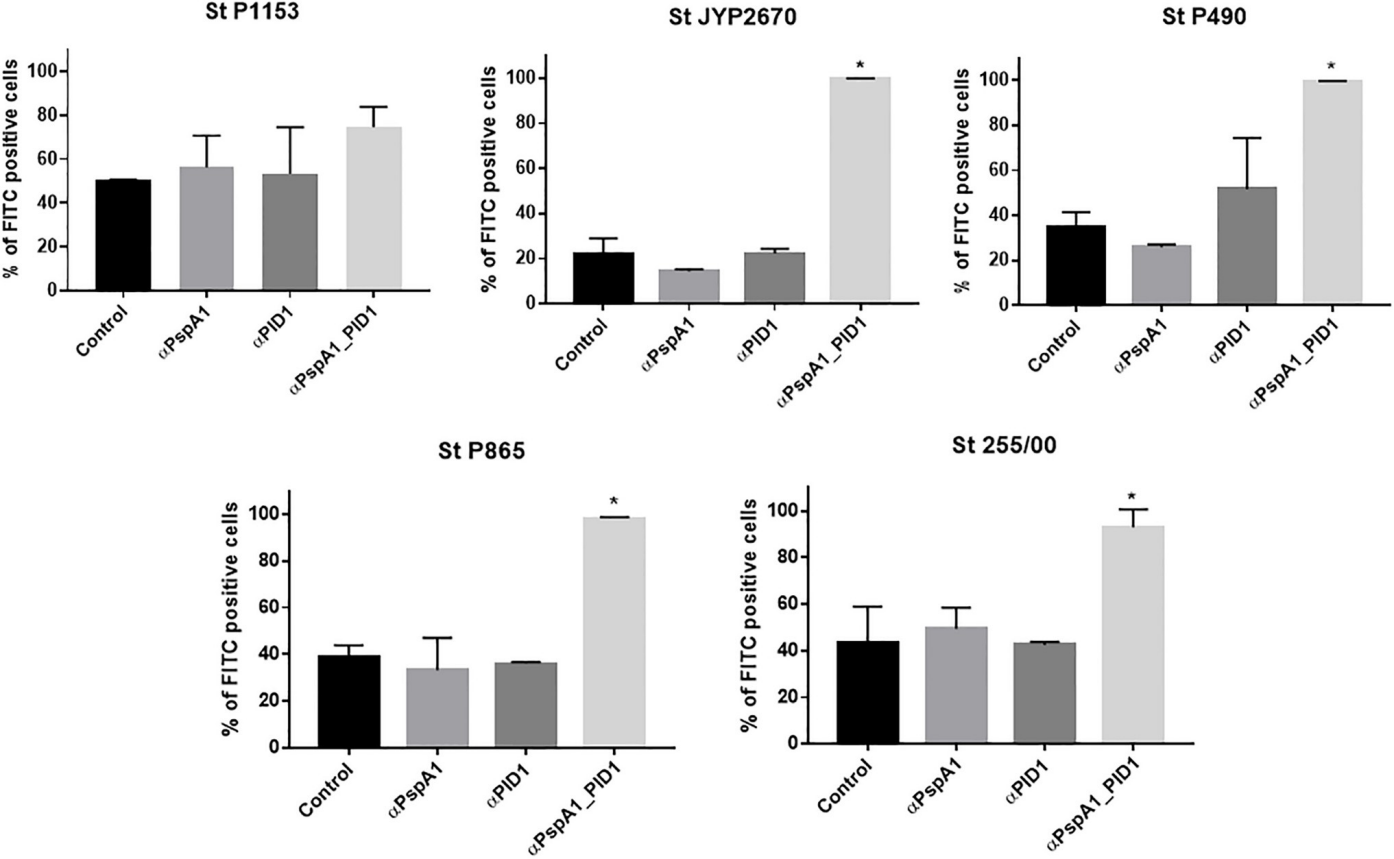
Complement deposition on the surface of *Streptococcus pneumoniae* in presence of specific antibodies. Pneumococcal strains St 255/00, 3JYP2670, P490, P865 and P1153 were treated with serum from mice immunized with PspA1, PlD1, the fusion PspA1_PlD1 or adjuvant alone (control), NMS as a complement source and FITC conjugated anti-mouse C3. The percentage of FITC positive bacteria (representing C3 binding) is shown for each group. The error bars represent the standard deviation of the replicates from the mean percentage of FITC positive cells. Statistical analysis was performed using ANOVA with Dunnet’s posttest. *p≤0,05 in comparison with the control.

Antibodies induced by immunization with the recombinant proteins were also evaluated for their capacity to promote complement C3 deposition onto the surface of pneumococci expressing heterologous PspA molecules. Five strains were used in the assay: one strain expressing a PspA of clade 3 (St P1153), two of clade 4 (3JYP2670 and P490) and two of clade 5 (St P865 and St 255/00). In accordance with the antibody binding results, sera from mice injected with PspA1_PlD1 induced an increase in the amount of C3 deposited on the surface of four out of five pneumococci tested, in comparison with the control (Fig 2). Furthermore, incubation with antibodies against the hybrid resulted in close to 100% positive cells in all but one pneumococcal strain. The only exception was the clade 3 strain P1153, which showed around 75% bacteria positive for C3 deposition against 50% in the control group and did not reach statistical significance. Meanwhile, similarly to the antibody binding results, antibodies against the isolated proteins did not promote complement activation and deposition on any of the strains tested.

**Fig 3:**
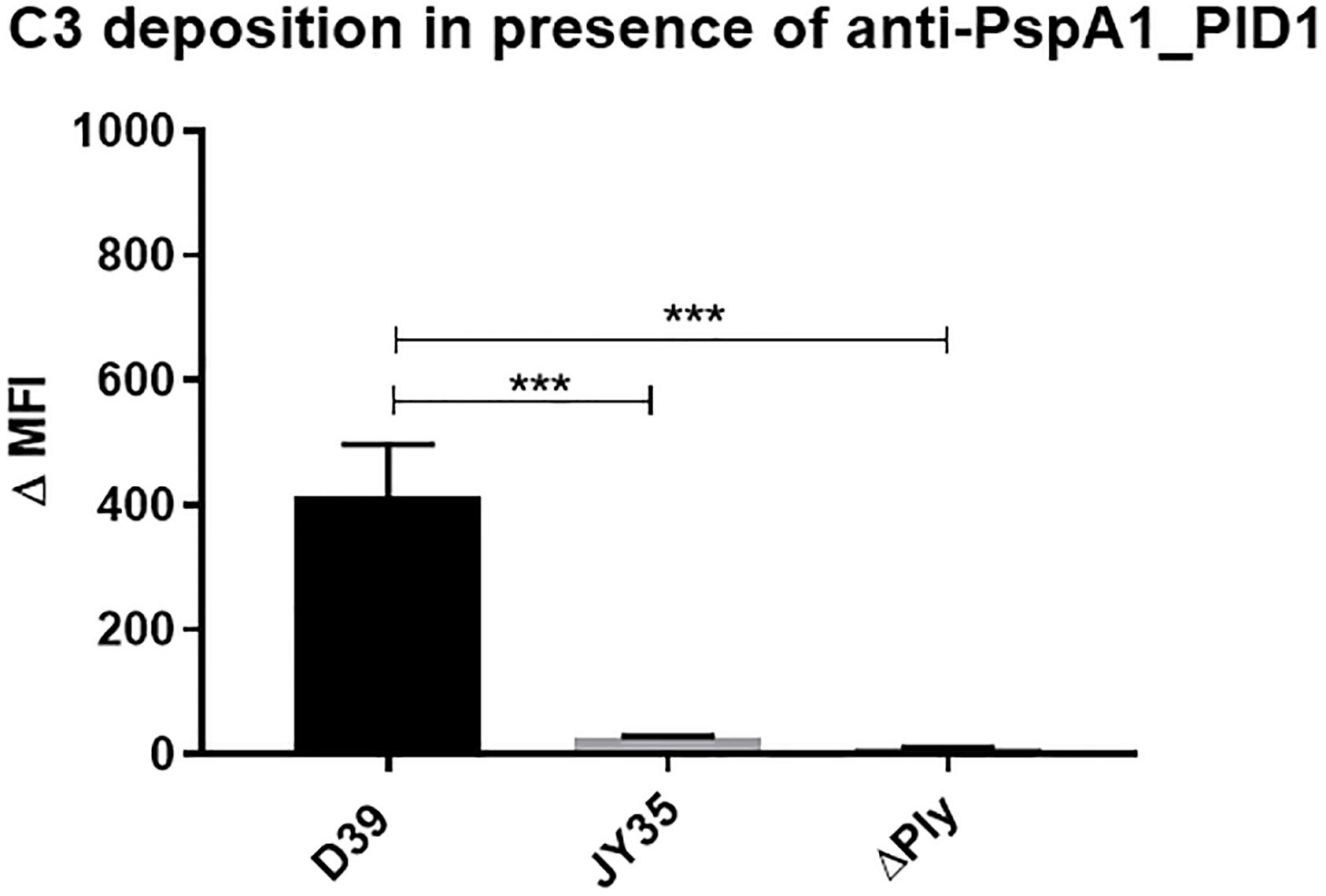
Comparison of antibody-induced complement deposition on wild-type and mutant *S*. *pneumoniae*. Pneumococcal strains D39 (wt), ΔPspA (PspA negative) and D39_ΔPly were incubated with anti-PspA1_PlD1 antisera or control sera (from sham immunized mice), NMS and FITC conjugated anti-mouse C3. The graph shows the ΔMFI (median of fluorescence intensity) of each sample subtracting the MFI of the control. The error bars represent the standard deviation of the replicates from the mean value. Statistical analysis was performed using ANOVA with Tuckey’s posttest. *p≤0,05 comparing wt and mutant bacteria.

To examine the contributions of each individual protein to complement deposition on pneumococci, the strain D39 and its isogenic mutants that do not express PspA or Ply were incubated with serum from mice which had been immunized with PspA1_PlD1. As shown in Fig 3, antibodies against the hybrid promoted higher levels of C3 deposition on the wild-type strain in comparison with the PspA^-^ and Ply^-^mutants (p<0,0001). The histograms showing the effect of antibodies in complement deposition are included in S2 Fig.

### Vaccine-induced protection against pneumococcal infection

**Fig 4:**
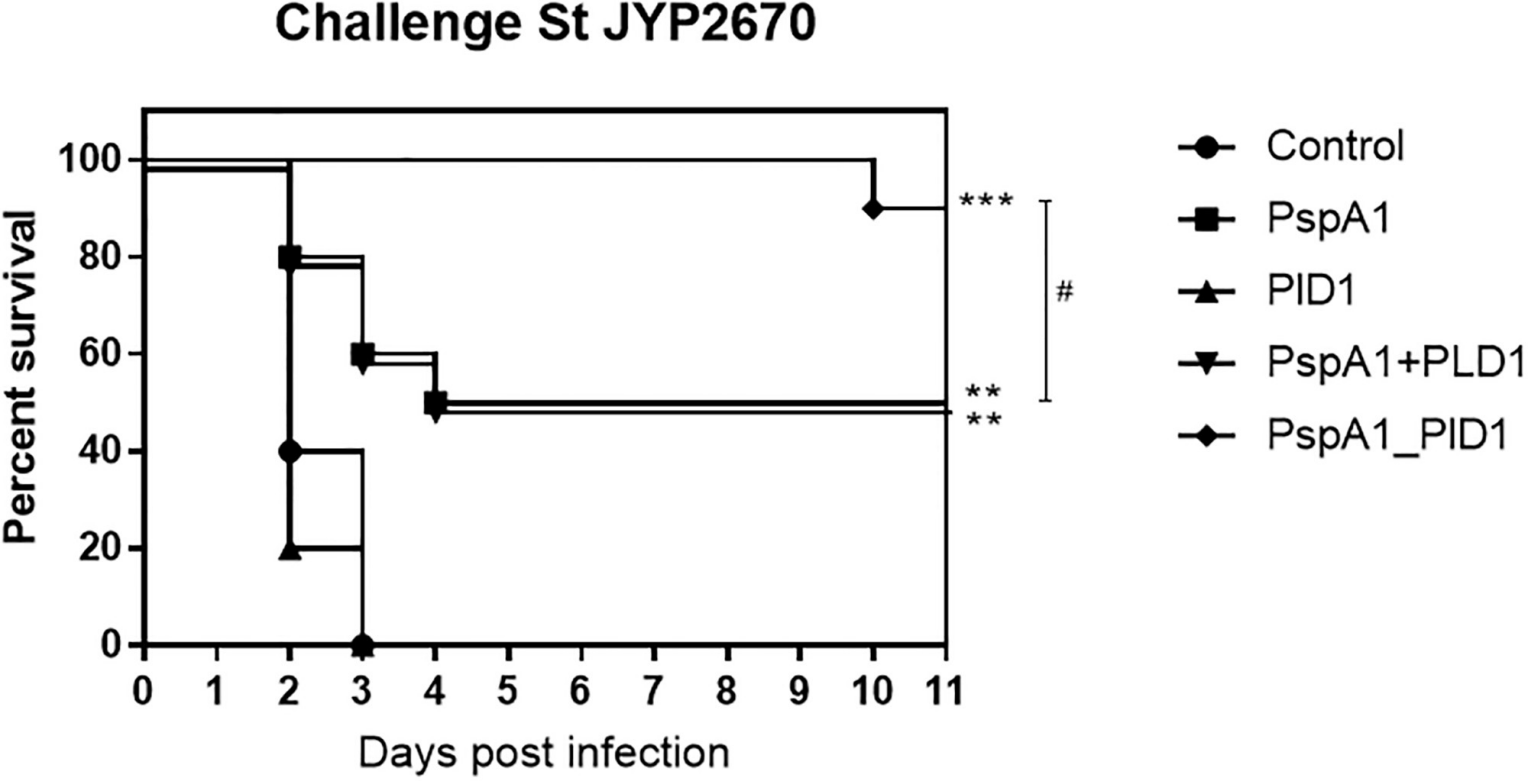
Survival of vaccinated mice after i.v. challenge with *S*. *pneumoniae*. Mice were immunized s.c. with three doses of PspA1, PlD1, the co-administered proteins or the PspA1-PlD1 hybrid, challenged i.v. with 10^6^ CFUs of *S*. *pneumoniae* strain 3JYP2670 and monitored for 12 days. The control group received adjuvant diluted in saline solution. Survival times are shown individually in the different immunization groups. Statistical analysis was performed using log rank analysis. *p≤0,05 in comparison with the control; ^#^ p≤0,05 when comparing different immunization groups.

For evaluation of cross-protection induced by the recombinant proteins, mice immunized with rPspA1, rPlD1, rPspA1_PlD1 fusion or the co-administered proteins were challenged intravenously with 10^6^ CFUs of the pneumococcal strain 3JYP2670, expressing a clade 4 PspA (Table 1). Mice injected with PBS and Al(OH)_3_ were used as a control. After three days of infection, all control mice had died (Fig 4). In contrast, the group immunized with PspA1_PlD1 fusion showed 90% survival after 12 days. PspA1 alone and the PspA1 + PlD1 mixture were also protective against infection; however, only 50% of the mice survived the challenge in each of these groups. Furthermore, when comparing the survival curves of the immunized mice, those immunized with the hybrid had longer survival times, with all mice alive until day 10. PspA and the protein mixture had similar survival curves, with 5 mice dead after four days of challenge. PlD1 alone did not confer protection against sepsis; all mice died within four days of infection.

## Discussion

PspA and Ply are two widely studied pneumococcal proteins displaying key roles in pneumococcal disease progression. Both proteins contribute to immune evasion and invasion of host tissues; Ply promotes cell damage and inflammation, while PspA limits opsonization and killing by complement molecules and antimicrobial peptides [5]. It can also bind to GAPDH on dying lung cells, facilitating bacterial dissemination during pneumonia [24].

For their important contributions to pneumococcal infections, PspA and Ply have been investigated as potential candidates in serotype-independent vaccines in several disease models [3, 25] and clinical trials [26]. The combination of PspA and pneumolysin derivatives has been particularly effective against severe outcomes like pneumonia [27] and bacteremia [8, 12, 28].

Previous work has shown that immunization with a PspA1-PlD1 chimera protects mice against systemic infection with virulent pneumococci bearing PspAs from family one [12]. However, PspA shows some level of variability, especially on the N-terminal, surface exposed region of the molecule (also known as the alfa-helical portion of the molecule), resulting in a mosaic-like sequence pattern. Since these variations impact the coverage of vaccines including PspA as an immunogen, we sought to evaluate the cross-protection induced by the rPspA1_PlD1 chimera against challenge with a pneumococcal strain bearing a heterologous PspA belonging to family 2. The construct included the N-terminal region plus the proline-rich domain of a clade 1 PspA molecule. Despite inducing similar IgG levels as each individual protein [12], the fusion protein was highly protective against this strain, with 90% of the mice surviving challenge. Interestingly, the co-administered proteins induced the same protection as PspA alone, indicating the fusion of PspA and Ply is important to increase the protective efficacy of this vaccine formulation. This could be due to modifications in the structure of the molecule resulting from the fusion, leading to exposure of protective epitopes. Although this hypothesis needs further investigation to be confirmed, a similar effect has been described for PspA in fusion with capsular polysaccharide 23F [29].

Next, we evaluated the mechanism underlying the increased protection observed with the rPspA-PlD1 chimera. Production of opsonic antibodies has long been identified as an important process for pneumococcal clearance in the host and a correlate of protection in current vaccines [30, 31]. Therefore, we tested if antibodies produced in response to immunization could bind to and increase complement deposition on bacteria bearing diverse PspAs. In accordance with the protection data, sera from mice injected with the rPspA-PlD1 hybrid showed increased binding to PspA family 2 strains, including the challenge isolate, 3JYP2670. Similarly, this antiserum led to higher levels of C3 deposition on the surface of different family two expressing pneumococci, in comparison with the individual proteins. In four out of five strains tested, the percentage of positive bacteria was close to 100%, attesting the ability of such antibodies to activate complement deposition and opsonization. Moreover, similar results were observed in bacteria producing different capsules, suggesting that the formulation is effective against multiple pneumococcal serotypes.

Finally, analysis of C3 deposition on mutant pneumococci lacking PspA or Pneumolysin showed a marked reduction when compared with the wild-type strain, indicating that both proteins contribute to the induction of opsonic and complement activating antibodies.

Anti-PspA antibodies have been shown to contribute to pneumococcal clearance through different mechanisms (reviewed in [32]), including increased complement deposition and opsonophagocytic killing by neutrophils [33] enhancing the bactericidal action of lactoferrin [34–36] and facilitating pneumococcal killing by neutrophil extracellular traps [37]. Although the present study only investigated complement activation by these antibodies, all these mechanisms could aid in preventing pneumococcal disease in vaccinated mice. Pneumolysin neutralization by antibodies prevent binding to cholesterol as well as its subsequent cytotoxic and platelet disrupting effects [38, 39]. Anti-ply antibodies also play a role in limiting biofilm formation and colonization by pneumococci [40–42]. Although immunization with PlD1 alone was not protective in the present study, the adjuvant capacity of pneumolysin derivatives could also contribute to the induction of amplified immune responses with higher cross-reactivity.

## Conclusion

The present study confirms the potential of rPspA1-PlD1 as a serotype independent pneumococcal vaccine and expands the previous results, demonstrating that the PspA-PlD1 fusion combines the protective traits of both proteins (i.e., the high immunogenicity of PspA and the conservation of Pneumolysin among pneumococci) inducing antibodies that efficiently promote complement deposition on multiple strains and cross-protection. The present data contributes to the development of serotype-independent, protein-based pneumococcal vaccines with high coverage, without the limitations of the current polysaccharide-based vaccines.

## Supporting information

S1 Fig Schematic representation of rPspA-PlD1. PspA1 N- and C-terminal domains are shown. The arrow in PlD1 marks the His_367_-Arg aminoacid replacement in the final protein. The chimeric protein includes the N-terminal domain of PspA1 fused to the complete PlD1 sequence.
(TIF)

S2 Fig Complement C3 deposition on wild-type and mutant pneumococci. D39 and its isogenic PspA^-^ (JY53) and Ply^-^ (D39_ΔPlt) mutant strains were incubated with sera from mice vaccinated with the hybrid protein, rPspA-PlD1, NMS and FITC-conjugated anti-mouse C3. The median fluorescence intensity (MFI) is shown for each bacterium.
(TIF)
