# Supplementary figures and images for: Fusion of PspA to detoxified pneumolysin enhances pneumococcal vaccine coverage
Source: PLoS One. 2023 Dec 14;18(12):e0291203. doi: 10.1371/journal.pone.0291203 (PMC10721071; doi:10.1371/journal.pone.0291203)

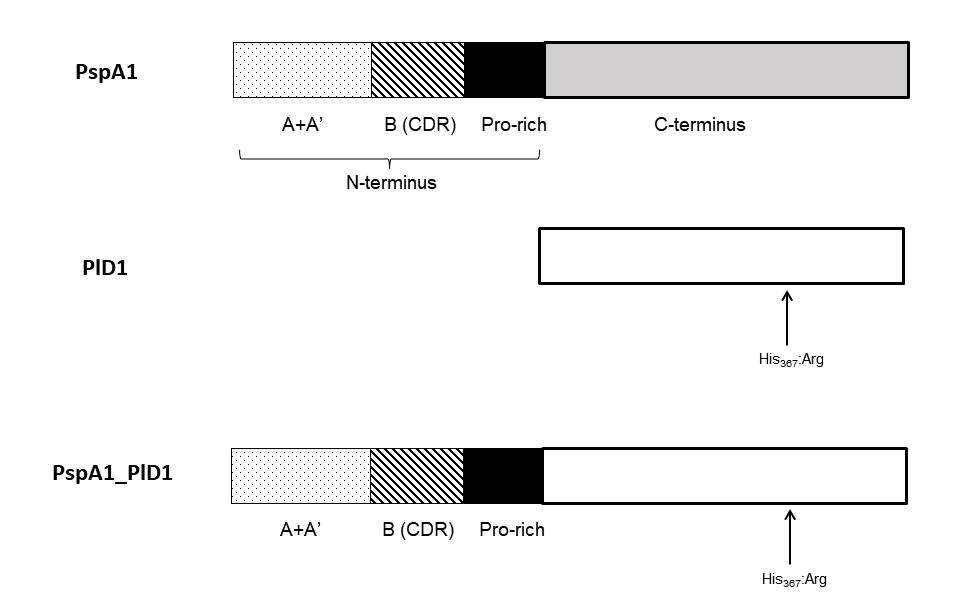

Supplement: S1 Fig — PspA1 N- and C-terminal domains are shown. The arrow in PlD1 marks the His367-Arg aminoacid replacement in the final protein. The chimeric protein includes the N-terminal domain of PspA1 fused to the complete PlD1 sequence. (TIF) [file pone.0291203.s001.tif]

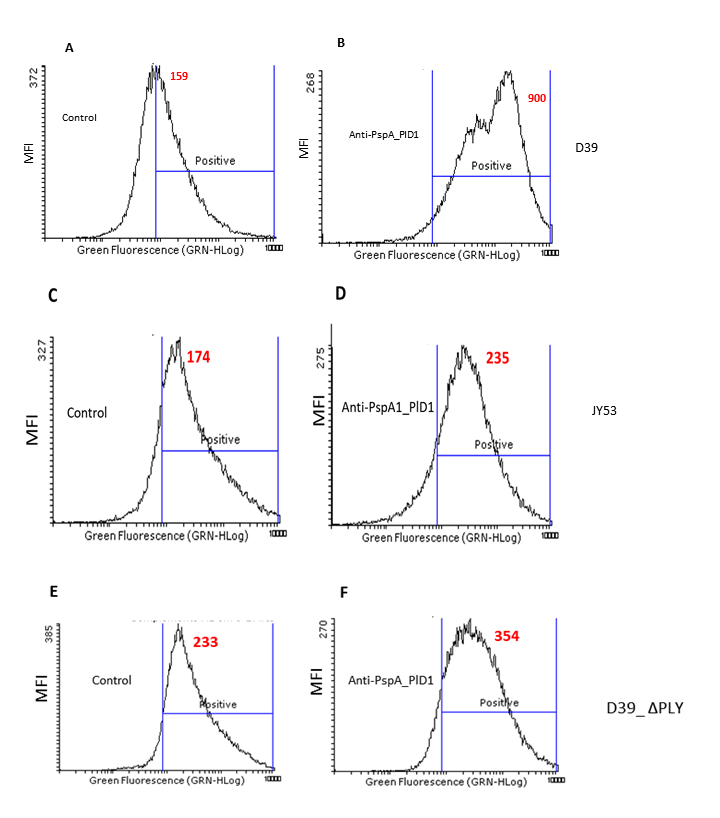

Supplement: S2 Fig — D39 and its isogenic PspA- (JY53) and Ply- (D39_ΔPlt) mutant strains were incubated with sera from mice vaccinated with the hybrid protein, rPspA-PlD1, NMS and FITC-conjugated anti-mouse C3. The median fluorescence intensity (MFI) is shown for each bacterium. (TIF) [file pone.0291203.s002.tif]
